# A Simple Algorithm Using Ventilator Parameters to Predict Successfully Rapid Weaning Program in Cardiac Intensive Care Unit Patients

**DOI:** 10.3390/jpm12030501

**Published:** 2022-03-21

**Authors:** Wei-Teing Chen, Hai-Lun Huang, Pi-Shao Ko, Wen Su, Chung-Cheng Kao, Sui-Lung Su

**Affiliations:** 1 Division of Thoracic Medicine, Department of Medicine, Cheng Hsin General Hospital, Tri-Service General Hospital, National Defense Medical Center, Taipei 112401, Taiwan; unirigin@gmail.com; 2 School of Public Health, National Defense Medical Center, Taipei 114201, Taiwan; ndmchelenhuang@gmail.com (H.-L.H.); kimirrarike@gmail.com (P.-S.K.); suwen9319@gmail.com (W.S.); 3 Institute of Aerospace and Undersea Medic, National Defense Medical Center, Taipei 114201, Taiwan; 4 Tri-Service General Hospital Songshan Branch, National Defense Medical Center, Taipei 105309, Taiwan; kao8267kq@gmail.com

**Keywords:** machine learning, ventilator weaning, weaning indicators, weaning success prediction

## Abstract

Background: Ventilator weaning is one of the most significant challenges in the intensive care unit (ICU). Approximately 30% of patients fail to wean, resulting in prolonged use of ventilators and increased mortality. There are numerous high-performance prediction models available today, but they require a large number of parameters to predict and are thus impractical in clinical practice. Objectives: This study aims to create an artificial intelligence (AI) model for predicting weaning time and to identify the most simplified key predictors that will allow the model to achieve adequate accuracy with as few parameters as possible. Methods: This is a retrospective study of to-be-weaned patients (*n* = 1439) hospitalized in the cardiac ICU of Cheng Hsin General Hospital’s Department of Cardiac Surgery from November 2018 to August 2020. The patients were divided into two groups based on whether they could be weaned within 24 h (i.e., “patients weaned within 24 h” (*n* = 1042) and “patients not weaned within 24 h” (*n* = 397)). Twenty-eight variables were collected including demographic characteristics, arterial blood gas readings, and ventilation set parameters. We created a prediction model using logistic regression and compared it to other machine learning techniques such as decision tree, random forest, support vector machine (SVM), extreme gradient boosting, and artificial neural network. Forward, backward, and stepwise selection methods were used to identify significant variables, and the receiver operating characteristic curve was used to assess the accuracy of each AI model. Results: The SVM [receiver operating characteristic curve (ROC-AUC) = 88%], logistic regression (ROC-AUC = 86%), and XGBoost (ROC-AUC = 85%) models outperformed the other five machine learning models in predicting weaning time. The accuracies in predicting patient weaning within 24 h using seven variables (i.e., expiratory minute ventilation, expiratory tidal volume, ventilation rate set, heart rate, peak pressure, pH, and age) were close to those using 28 variables. Conclusions: The model developed in this research successfully predicted the weaning success of ICU patients using a few and easily accessible parameters such as age. Therefore, it can be used in clinical practice to identify difficult-to-wean patients to improve their treatment.

## 1. Introduction

Ventilator weaning is one of the most significant challenges in the ICU. Thirty percent of patients fail to wean, resulting in prolonged use of ventilators and increased mortality [1]. Each year, more than one million patients in the USA require ventilators and spend up to USD 27 billion on them [2,3]. Long-term ventilation increases the risk of complications such as ventilator-associated pneumonia [4], so patients should be weaned as soon as possible. Three of the previous studies in thee literature are concerned with artificial neutral network (ANN)-based models. Kuo et al. gathered eight ventilation set parameters to predict weaning outcomes with a receiver operating characteristic curve (ROC-AUC) of 83% [5]. Hsieh et al. selected 37 ventilation set parameters to predict weaning outcomes with a ROC-AUC of 85% [6]. Hsieh et al. used 47 ventilation set parameters to predict simple, difficult, or prolonged weaning with a ROC-AUC of 84.9–94.2% [7]. Otaguro et al. used various models such as LightGBM as well as 57 ventilation set parameters to predict weaning outcomes with a ROC-AUC of 95% [8]. However, these studies used a large number of parameters for prediction, which are difficult to collect in clinical practice, making it difficult to create an available model or decreasing the model’s accessibility. A model with more parameters is more complicated. When developing a model, we hope to identify the appropriate parameters that will aid in the interpretation and prediction of test data. Although using a large number of parameters can sometimes improve a model’s performance, some parameters may be superfluous, affecting the model’s output and lowering its interpretability, or may not help improve its prediction performance. Hence, we need to thoroughly assess and select the parameters that affect the model most significantly [9]. Currently, several high-performance prediction models are available, but they require numerous parameters for prediction and are thus impractical in clinical practice. This study aimed to develop a simple artificial intelligence (AI) model that uses a few parameters and identifies the most simplified key predictors that will allow the model to achieve adequate accuracy with as few parameters as possible for predicting weaning time.

## 2. Materials and Methods

### 2.1. Study Design and Setting

**Figure 1:**
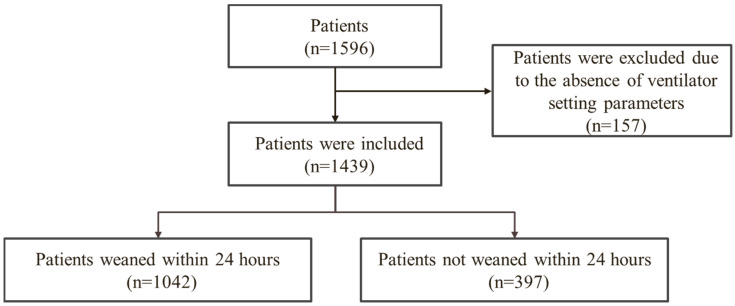
Study flowchart.

As shown in Figure 1, this research is a retrospective study, which was approved by the Institutional Review Board under approval number A202006127, on the patients (n = 1439) (absence of ventilator setting parameters are excluded) who were mechanically ventilated in the cardiac intensive unit of the Department of Cardiac Surgery, Cheng Hsin General Hospital, from November 2018 to August 2020. The patients are divided into two groups based on whether they could be weaned within 24 h (i.e., “patients weaned within 24 h” (*n* = 1042) and “patients not weaned within 24 h” (*n* = 397)). This research analyzed whether the patient could be weaned within 24 h.

There was a total of 1596 patients in the cardiac intensive unit of the Department of Cardiac Surgery. After excluding 157 patients who did not have ventilator setting parameters, 1439 patients remained. Patients were divided into two groups based on whether or not they could be weaned within 24 h: “patients weaned within 24 h” (*n* = 1042) and “patients not weaned within 24 h” (*n* = 397).

### 2.2. Data Source

We collected 28 variables from the ICU cases: demographic variables including gender, age, and smoking; physiological variables including systolic blood pressure, diastolic blood pressure, and heart rate; ventilation set parameters including ventilation rate set, inspiration time, pressure limit high, spontaneous respiratory rate, inspiratory pressure, PEEP, ramp, pressure limit low, inspiratory tidal volume, expiratory tidal volume, peak pressure, mean pressure, expiratory minute ventilation, compliance, and resistance; and arterial blood gas (ABG) readings including SpO_2_, pH, PCO_2_, HCO_3_, PO_2_, SAO_2_, and base excess. Demographic information was derived from case records, physiological and ventilation set variables were derived from respiratory therapy records, and ABG readings were derived from the laboratory. The ventilators were classified as Evita 4, Evita XL, and Evita 2 Dura.

### 2.3. Algorithms

To predict the weaning outcome, this paper used six machine learning techniques including logistic regression (LR), decision tree (DT), random forest, extreme gradient boosting (XGBoost), support vector machine (SVM), and ANN.

#### 2.3.1. Logistic Regression (LR)

LR is similar to the linear one, but it is mostly used for classification instead of regression tasks. To predict the likelihood of a data point belonging to a class, LR fits a sigmoid curve to the training inputs. It is referred to as a regression because it predicts the likelihood rather than the class directly [10]. This method employs the following hyperparameters: loss function = L2, cost function = 0.02, and epsilon = 0.001.

#### 2.3.2. Decision Trees (DT)

Decision trees are supervised algorithms designed to find a path to the target variable using a set of decisions from the input variables. They can either be continuous (regression trees) or categorical (classification trees). As a result, the algorithm is known as classification and regression tree. Because decision trees are similar to decision charts and are thus easy to interpret, they are commonly used as first-step algorithms when approaching new problems. However, they are prone to overfitting the data and do not generalize well to novel situations and datasets [11]. This method employs the following hyperparameter: CP denotes the tree’s complexity parameter, which is set to 0.

#### 2.3.3. Random Forest (RF)

This is an ensemble method that combines multiple decision trees into a final decision. Because individual decision trees tend to overfit, random forests mitigate individual biases by combining and weighting the outputs of multiple decision trees (regression or classification) [12]. The hyperparameters used in this method are as follows: the number of predictors randomly sampled at each split (mtry) = 4, the maximum number of nodes (maxnode) = 20, and the method used to build the largest tree structure = Gini classification method.

#### 2.3.4. eXtreme Gradient Boosting (XGBoost)

Gradient boosting is a machine learning technique for regression and classification problems, producing a prediction model in the form of an ensemble of weak prediction models (typically decision trees). It constructs the model in a stage-wise manner, similar to other boosting methods, and generalizes them by allowing optimization of an arbitrary differentiable loss function. Extreme gradient boosting (XGBoost) is a scalable and improved version of the gradient boosting algorithm (terminology alert) designed for efficacy, computational speed, and model performance [13]. The hyperparameters used by this method are as follows: the number of trees (or rounds) in the model (nrounds) = 10, the maximum depth of the tree model (max_depth) = 2, the learning rate (eta) =0.3, gamma = 0.1, subsampling for determining the percentage of the raw training dataset used = 0.75, the number of columns used by each tree (colsample_bytrees) = 0.9, and the minimum sum of weights of all observations required in a child (min_child_weight) = 2.

#### 2.3.5. Support Vector Machine (SVM)

SVMs are extremely powerful supervised statistical modeling algorithms used for both regression and classification. In classification tasks, SVMs find a hyperplane (imagine a plane in a new dimension) between different classes of data, which is later used with novel examples to classify them [14]. The hyperparameters used by this method are as follows: type = classification, the large C in the Lagrange formulation, which determines the penalty value for the error/division data, and cost C = 1.

#### 2.3.6. Artificial Neural Network (ANN)

Artificial neural network is a family of algorithms covering classification tasks, regression tasks, ensemble tasks, and feature discovery. The basic architecture is consistent across implementations: the algorithm is divided into multiple layers, beginning with the input layer (where input examples are represented) and ending with the output layer (where the resulting regression/classification/ensemble is represented), with optional (hidden) layers in between [15]. The hyperparameters used by this method are size = 10 and decay = 0.2.

In ML, supervised, unsupervised, semi-supervised, and reinforcement learning are four common learning methods for solving different tasks. Among them, supervised learning is the most accurate but also the most labor-intensive. The advantage is that it can be trained precisely in the process. Therefore, the following common supervised learning models (LR, DT, RT, XGBoost, SVM, and ANN) are used for subsequent comparisons to develop the models to predict the weaning success of ICU patients.

### 2.4. Statistical Analyses

For descriptive statistics, the categorical variables are “frequency distribution” and “proportion,” and the continuous variables are “mean” and “standard deviation.” For inferential statistics, the independent *t*-test and chi-square test are used. The ROC is the primary predictor, with the true positive rate (TPR) as the *x*-axis and the false positive rate as the *y*-axis. The higher the TPR and the better the accuracy, the closer the curve is to the top. The greater the area under the ROC curve (ROC-AUC), the better the model. The ROC-AUC scale runs from 0 to 1, with 1 being the best value.

#### 2.4.1. Features Extraction

Forward, backward, and stepwise selection methods are used to select significant variables [16]. The forward selection method adds independent variables one by one to the model until the contribution of any independent variable is no longer statistically significant. The backward selection method removes independent variables from the model one by one until the model loses too much explanatory power when any independent variable is removed. The stepwise selection method is a hybrid of the forward and backward selection methods, and it differs in that it can both add excluded variables to the model and delete selected variables from the model.

#### 2.4.2. Analysis Procedure

**Figure 2:**
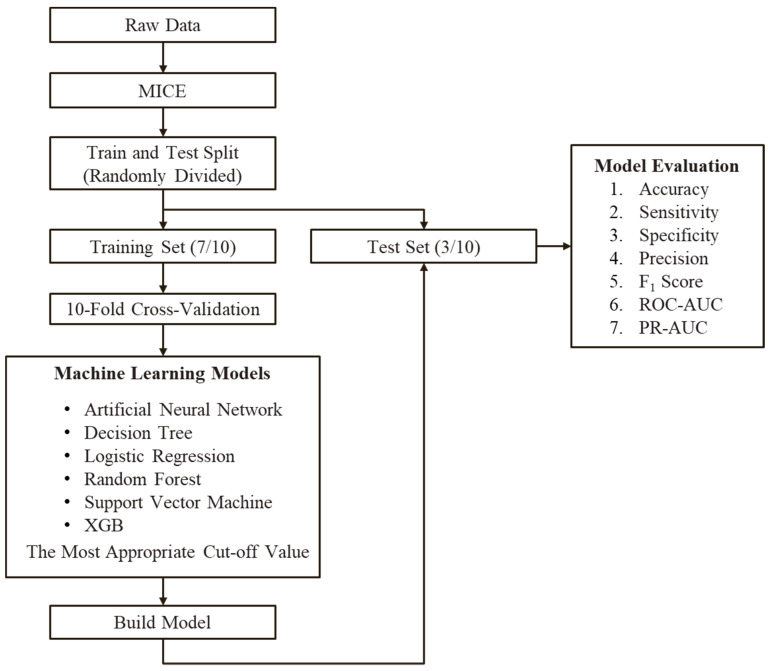
Flowchart of the proposed method.

As shown in Figure 2, after the raw data are input, the Multivariate Imputation by Chained Equations (MICE) package is used to fill the missing values to create a complete dataset. The dataset is then divided into two parts: training and testing. For ten-fold cross-validation, the training dataset is divided into 10 equal parts. Part 1 is used as the validation test data, and the remaining nine parts are used for training. Part 2 is used as test data for validation in the next round, and the remaining nine parts are used for training. This procedure is repeated ten times. For prediction, six models (artificial neural network, DT, LR, random forest, SVM, and XGBoost) were used. The results obtained after modifications were used to create models, which were tested with the test dataset. In the end, seven indicators (accuracy, sensitivity, specificity, precision, F_1_ score, ROC-AUC, and PR-AUC) for machine learning models were used to evaluate the results of the six models.

After the raw data were input, the MICE package was used to fill the missing values to create a complete dataset. The dataset was then split into the training and test datasets. The test dataset was used to predict six models (artificial neural network, decision tree, logistic regression, random forest, support vector machine, and XGBoost). The modified results were used to create models, which were tested using the test dataset. Finally, seven machine learning model indicators (accuracy, sensitivity, specificity, precision, F1 score, ROC-AUC, and PR-AUC) were used to evaluate the results of the six models.

MICE: Multivariate Imputation by Chained Equations

ROC-AUC: Receiver operating characteristic curve area under the curve

PR-AUC: Precision–recall curve area under the curve

## 3. Results

### 3.1. Patient Characteristics

Table 1 lists the demographic, physiological, ventilation set, and ABG data of the two groups. Among the demographic variables, there were significant differences in age and smoking (*p*-value < 0.001). There were significant differences in all the physiological data (*p*-value < 0.001) and ventilation set data (*p*-value < 0.001). In addition, there were significant differences in all ABG data except pH.

### 3.2. Results of the Machine Learning Models

**Figure 3:**
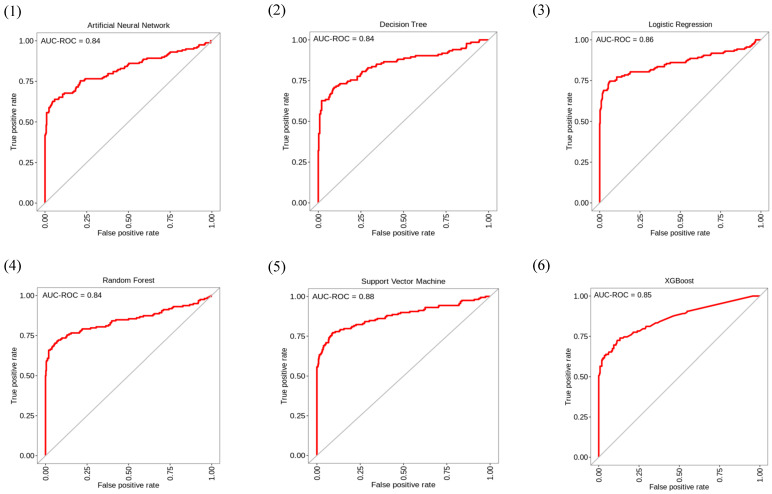
Receiver operating characteristic curves for each of the six machine learning methods. ROC-AUC of the six models [(**1**) artificial neural network, (**2**) decision tree, (**3**) logistic regression, (**4**) random forest, (**5**) support vector machine, and (**6**) XGBoost] for predicting patient weaning within 24 h. ROC-AUC: Receiver operating characteristic curve area under the curve.

**Figure 4:**
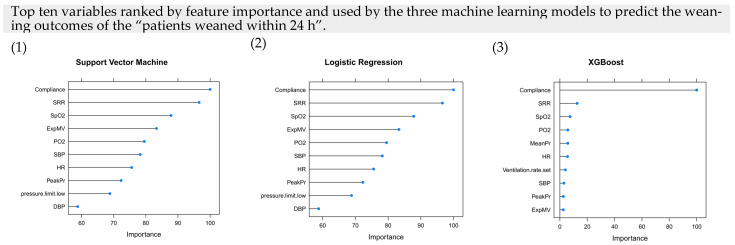
Feature importance for the machine learning methods in the proposed algorithm. Top ten variables ranked by feature importance and used by the three machine learning models [(**1**) support vector machine, (**2**) logistic regression, and (**3**) XGBoost] to predict patient weaning within 24 h. Abbreviations: *SRR* spontaneous respiratory rate, *Exp MV* expiratory MV, *Exp TV* expiratory TV, *InspTV* inspiratory tidal volume, *HR* heart rate, *PeakPr* peak pressure, *MeanPr* mean pressure, *DBP* diastolic blood pressure, *SBP* systolic blood pressure.

Figure 3 and Table 2 show the ROC-AUC of the six machine learning models and the results of their seven corresponding indicators. The top three performers among the six machine learning models were the SVM, LR, and XGBoost models. The ROC-AUC values for the SVM, LR, and XGBoost models for predicting patient weaning within 24 h were 88%, 86%, and 85%, respectively, and were not significantly different from one another. Figure 4 shows the feature importance of variables for the three machine learning models.

The top three variables ranked by feature importance and used by the SVM, LR, and XGBoost models to predict patient weaning within 24 h were compliance, spontaneous respiratory rate (SRR), and SpO_2_.

### 3.3. Using LR to Create a New Prediction Model

**Figure 5:**
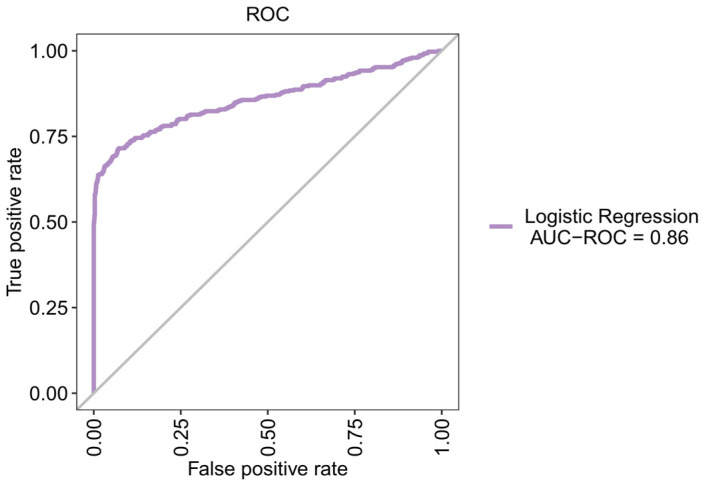
ROC-AUC for a NEW logistic regression model. ROC-AUC for predicting patient weaning within 24 h. ROC-AUC: Receiver operating characteristic curve area under the curve.

As shown in Table 3, we used LR to create a new model and the forward, backward, and stepwise selection methods to select seven significant predictors (i.e., expiratory minute ventilation (ExpMV), expiratory tidal volume (ExpTV), ventilation rate set (VenRS), heart rate (HR), peak pressure (PeakPr), pH, and age). As shown in Figure 5, the ROC-AUC, sensitivity, and specificity values of the new LR model for predicting patient weaning within 24 h were 86%, 64%, and 98.2%, respectively. These values were close to those when 28 predictors were used.

The following equation is established based on the above seven parameters with the cutoff value being 0:

Score=−11.430+0.397 ∗ ExpMV−0.010 ∗ ExpTV+0.094 ∗ VenRS+0.017 ∗ HR+0.069∗ PeakPr−0.667 ∗ pH+0.015 ∗ age

For example, there is a 69-year-old patient with *ExpMV* of 17 cmH_2_O, *ExpTV* of 390 cmH_2_O, *VenRS* of 20 30/min, *HR* of 104 bpm *PeakPr*, of 24 cmH_2_O, and pH of 7. To predict weaning outcomes of the patients weaned within 24 h, we shall substitute these values into the equation to calculate the values of Score, which is −6.91.

Score=−11.430+0.397 ∗ 17−0.010 ∗ 390+0.094 ∗ 20+0.017 ∗ 104+0.069 ∗ 24−0.667 ∗7+0.015 ∗ 69=−6.91

## 4. Discussion

This research found out that the SVM, LR, and XGBoost models using 28 variables efficiently predicted weaning outcomes. In addition, we used LR to establish simple equations for predicting weaning outcomes and selected seven common and easily accessible variables. The ROC-AUC values of this new model for predicting patient weaning within 24 h was 86%, which was as good as those of the models using 28 variables.

The ROC-AUC values of the model created in this research ranged from 85% to 91%, which were similar to that of 83% of the model created by Kuo et al. to predict weaning outcomes using eight ventilation set parameters [5], 85% of the model created by Hsieh et al. in 2019 for predicting weaning outcomes using 37 ventilation set parameters and lab values [6], or 84.9–94.2% of the model created by Hsieh et al. in 2020 for predicting weaning outcomes using 47 ventilation set parameters and lab values [7]. Hence, the model created in this research could effectively predict weaning outcomes.

On the other hand, Otaguro et al. used various models such as LightGBM as well as 57 ventilation set parameters to predict weaning outcomes with a ROC-AUC of 95% [8], and Fabregat et al. used the gradient boosting method and SVM models as well as 20 ventilation set parameters and lab values to predict weaning outcomes with ROC-AUCs of 96.1% and 98.3%, respectively [17]. They performed better than the model described in this paper, possibly because they used more variables for prediction and can therefore predict weaning outcomes more accurately.

We observed that the previous models that could better predict weaning outcomes usually require numerous parameters for effective predictions and are hence complicated. Furthermore, gathering all of the parameters is difficult, limiting the models’ accessibility and making them impractical in clinical practice [5,6,7,8]. As a result, we chose seven variables, namely, ExpMV, ExpTV, VenRS, HR, peak pressure (PeakPr), pH, and age [18,19,20,21,22,23,24], and used the less complicated LR method to create a simple but effective model for predicting weaning outcomes. It will be simpler to incorporate this model into clinical practice.

As a retrospective study on cases of illness, this research had some limitations. Because the integration levels of various information systems across the hospital are different, we only collected limited variables and did not consider the significant variables mentioned in all studies (e.g., the Rapid Shallow Breathing Index, respiratory rate, and APACHE II score [19,21,22,24,25,26,27,28,29,30,31,32,33,34,35]). Cardiac variables such as left ventricular ejection fraction can be collected, which may make determining the severity of heart diseases impossible. This study focused on cardiac surgery. Patients who do not normally have lung diseases are mechanically ventilated only briefly after surgical procedures and thus have a lower risk of weaning failure, which may cause the results to be overestimated. Because this study only used data from one hospital, it cannot be applied to other medical institutions. Data from other hospitals can be used in the future to validate the model’s robustness and prediction performance.

## 5. Conclusions

The SVM, LR, and XGBoost models using 28 variables could efficiently predict weaning outcomes. The model developed in this research succeeded in effectively predicting the weaning success of ICU patients using seven common and easily accessible parameters. The ROC-AUC value of this new model for predicting patient weaning within 24 h was 86%, which was as good as those of the models using 28 variables. As a result, it can be used in clinical practice to identify and treat difficult-to-wean patients. However, data from other hospitals can be used in the future to validate the model’s robustness and prediction performance. In the future, Rapid Shallow Breathing Index, respiratory rate, APACHE II score, and cardiac variables such as left ventricular ejection fraction can be collected, and data from other hospitals can be used to verify the robustness and predictive performance of the model. When this model is implemented in clinical practice, its quantified values will assist doctors in making well-informed decisions more quickly, reducing the burden on patients, their families, medical resources, and society.

## Figures and Tables

**Table 1:** Demographic and clinical characteristics of 1439 cardiac intensive care unit patients with planned extubation.

Variable	Weaned within 24 h (*n* = 1042)	Not Weaned within 24 h (*n* = 397)	*p*-Value	
Gender			0.069	
Males	714 (68.5%)	252 (63.5%)		
Females	328 (31.5%)	145 (36.5%)		
age, mean ± SD	65.05 ± 12.53	68.34 ± 15.18	<0.001	*
Smoking, *n* (%)			<0.001	*
No	805 (77.3%)	250 (63.0%)		
Yes	198 (19.0%)	113 (28.5%)		
Yes, quit smoking	39 (3.7%)	34 (8.6%)		
Ventilation set, mean ± SD				
Ventilation rate set, 30/min	12.23 ± 1.08	14.77 ± 8.77	<0.001	*
Inspiration time, breath/min	1.00 ± 0.00	4.08 ± 8.72	<0.001	*
Pressure limit high, cmH_2_O	40.72 ± 1.89	37.00 ± 6.75	<0.001	*
Pressure limit low, cmH_2_O	2.99 ± 0.40	3.91 ± 1.10	<0.001	*
Spontaneous respiratory rate, %	13.38 ± 2.92	20.22 ± 6.45	<0.001	*
Inspiratory pressure, cmH_2_O	20.74 ± 2.52	19.69 ± 5.84	<0.001	*
PEEP, cmH_2_O	5.47 ± 0.92	4.37 ± 2.69	<0.001	*
Ramp, mS	0.01 ± 0.13	0.24 ± 0.73	<0.001	*
Ventilation monitoring, mean ± SD				
Inspiratory tidal volume, mL/kg	554.92 ± 84.93	422.41 ± 336.91	<0.001	*
Expiratory tidal volume, mL/kg	555.55 ± 80.85	507.98 ± 156.23	<0.001	*
Peak pressure, cmH_2_O	21.05 ± 2.85	211.52 ± 273.68	<0.001	*
Mean pressure, cmH_2_O	8.75 ± 1.33	13.37 ± 7.38	<0.001	*
Expiratory minute ventilation, L/min	7.26 ± 1.72	10.49 ± 4.13	<0.001	*
Compliance, mL/cmH_2_O	60.04 ± 29.42	28.78 ± 35.06	<0.001	*
Resistance, mL/cmH_2_O	13.68 ± 5.43	28.95 ± 33.67	<0.001	*
Arterial blood gas test, ABG, mean ± SD				
SpO_2_, %	99.36 ± 31.84	63.59 ± 36.53	<0.001	*
pH	7.03 ± 0.18	7.04 ± 0.18	0.947	
PCO_2_, mmHg	37.33 ± 8.15	32.03 ± 11.89	<0.001	*
HCO_3_, mmol/L	23.57 ± 4.06	29.36 ± 11.29	<0.001	*
PO_2_, mmHg	162.86 ± 96.22	72.75 ± 102.14	<0.001	*
SAO_2_, %	154.74 ± 140.22	230.17 ± 172.74	<0.001	*
Base Excess, mmol/L	2.99 ± 21.32	36.83 ± 49.32	<0.001	*
Others, mean ± SD				
Systolic blood pressure, mmHg	124.55 ± 24.57	97.67 ± 33.42	<0.001	*
Diastolic blood pressure, mmHg	66.50 ± 26.76	93.78 ± 41.28	<0.001	*
Heart rate, bpm	82.42 ± 14.98	106.87 ± 28.78	<0.001	*

*: *p*-value < 0.05.

**Table 2:** Performance comparisons of six machine learning methods.

Model	Accuracy	Sensitivity	Specificity	Precision	F_1_ Score	ROC-AUC	PR-AUC
Artificial neural network	85.2%	67.5%	91.7%	75.7%	71.4%	84.0%	76.0%
Decision tree	87.7%	66.2%	93.6%	79.7%	72.4%	84.0%	79.0%
Logistic regression	83.1%	64.5%	98.3%	93.4%	76.3%	86.0%	84.0%
Random forest	86.8%	67.5%	91.7%	75.7%	71.4%	84.0%	76.0%
Support vector machine	86.8%	64.2%	98.8%	95.5%	76.8%	88.0%	70.0%
XGBoost	85.8%	62.7%	98.6%	94.3%	75.3%	85.0%	82.0%

Seven indicators (accuracy, sensitivity, specificity, precision, F_1_ score, ROC-AUC, and PR-AUC) for machine learning models were used to evaluate the results of the six models (artificial neural network, decision tree, logistic regression, random forest, support vector machine, and XGBoost). ROC-AUC: Receiver operating characteristic curve area under the curve. PR-AUC: Precision–recall curve area under the curve.

**Table 3:** Logistic regression coefficients of seven variables.

Variable	Coefficient
Expiratory minute ventilation (L/min)	0.397
Expiratory tidal volume (mL/kg)	−0.010
Ventilation rate set (30/min)	0.094
Heart rate (bpm)	0.017
Peak pressure (cmH_2_O)	0.069
pH	0.667
Age	0.015
Intercept	−11.430

Logistic regression coefficients of seven variables for the groups with the cutoff value being 0.

## Data Availability

Not available.
